# Assessing the Premature Aging of Chabazite in Natural Gas Drying by TSA

**DOI:** 10.1021/acsomega.5c11701

**Published:** 2026-03-11

**Authors:** Pedro A. S. Moura, Elena Rodríguez-Aguado, Débora A. S. Maia, Juan A. Cecília, Darley C. Melo, Susana Valencia, Fernando Rey, Moises Bastos-Neto, Enrique Rodríguez-Castellón, Diana C. S. Azevedo

**Affiliations:** † Department of Chemical Engineering, Campus do Pici, bl. 731, Federal University of Ceara (UFC), Fortaleza, CE 60760-400, Brazil; ‡ Department of Inorganic Chemistry, Crystallography and Mineralogy, Campus de Teatinos, Universidad de Málaga, Málaga 29071, Spain; § CENPES/Petrobras, 21941-915 Rio de Janeiro, Brazil; ∥ Instituto de Tecnología Química, Consejo Superior de Investigaciones Científicas−Universitat Politècnica de València, 46022 Valencia, Spain

## Abstract

Ongoing research on adsorbent deactivation in drying processes has unveiled crucial insights into the loss of performance with prolonged use. Our study builds on previous work by examining the effects of high temperatures, hydrocarbon exposure, and cycle duration on chabazite adsorbents with a Si/Al ratio of approximately 2 and in the sodium form. By aging the material under controlled conditions, we assessed the impact on its structural and textural properties. Our findings reveal that maintaining high temperatures, coupled with exposure to *n*-heptane vapor, induces a mild degradation of the crystalline structure, with more pronounced effects during longer aging periods. Notably, hydrocarbons play a critical role in adsorbent deactivation, adsorbing in both the inner pores and the outer surface of the zeolite. This leads to a deterioration of the textural characteristics, which directly correlates with an increase in the carbon content of the bulk material. Additionally, samples exposed to *n*-heptane vapor exhibited a more homogeneous composition compared to those subjected to manual liquid *n*-heptane addition. Overall, the degree of degradation varies among aged samples, indicating the need for tailored applications based on specific aging conditions.

## Graphical abstract

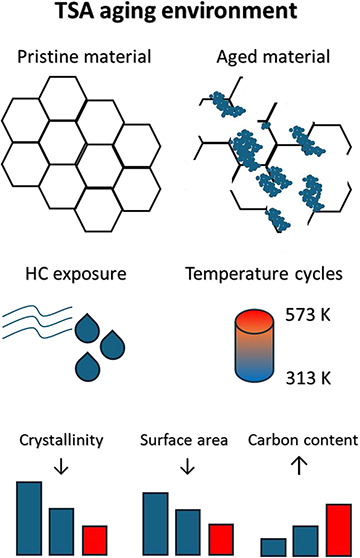

## 1 Introduction

The presence of water in natural gas poses a significant challenge to its widespread use. At sufficiently low temperatures, water vapor can form hydrates that trap hydrocarbons (C*_n_* H_2*n*_ + 2 × H_2_O) in a crystalline framework. These newly formed crystals can clog pipelines and equipment.^1^ Additionally, the water content reduces the calorific value of natural gas, reducing combustion efficiency. Moreover, water can react with carbon dioxide and hydrogen sulfide, potentially causing corrosion in equipment and pipelines.^2^ In this way, gas drying is a mandatory process, occasionally applied by Temperature Swing Adsorption (TSA). Summarizing, adsorbents used in gas drying by TSA processes must exhibit several key properties: they must be predominantly microporous to accommodate the small size of gas molecules, with narrow pores that enable significant adsorption. A large surface area is essential to maximize adsorption capacity, resulting in more compact adsorbents. Microporous adsorbents must also possess good mechanical strength and an appropriate particle size to minimize pressure drops in adsorption columns. Additionally, they must selectively adsorb the target adsorbate (water vapor molecules) and distinguish it from other gases. Furthermore, good thermal resistance is crucial for enduring the thermal swings encountered during the process.

Zeolites are crystalline structures with well-organized and specific pore sizes, making them ideal for the separation of small molecules such as carbon dioxide and water. Chabazite materials (CHA) are a type of molecular sieve found in natural mineral deposits or produced synthetically. This small-pore zeolite features 8-membered ring pores (0.38 × 0.38 nm^2^) that form channels within a rhombohedral crystal structure.^3,4^ The CHA framework can readily accommodate various exchange cations, giving rise to cationic zeolites. These cations include Na, K, Ca, Mg, Ba, among others,^5,6^ and can be located at three possible sites within the structure.

The hydrothermal stability of CHA materials is significantly influenced by exchange cations and can be summarized in five key features that interact in the context of adsorption. The aluminum content in CHA materials is governed by Löwenstein’s rule, which prohibits Al–O–Al bonds in zeolites.^7^ Aluminum coordination can change from tetrahedral (in the zeolite framework) to octahedral (extra-framework) even at room temperature.^8^ This change affects the material’s acidity, which can manifest as Brønsted or Lewis acid sites.^9,10^ Brønsted acidity is associated with bridged hydroxyl groups, the strongest acidic sites in zeolites, formed by protons (H^*+*^) attracted to negative framework oxygens linked to Al and Si atoms. Extra-framework aluminum (EFAl) acts as a Lewis acid site,^11^ capable of accepting electron pairs. Cationic zeolites typically exhibit more Lewis acid sites due to EFAl and structural defects.^12^ Silicon content affects the thermal resistance due to the stability of Si–O–Si bonds. High silicon content requires more energy to break these bonds, contributing to higher thermal stability.^13,14^ The polarity of the materials, governed by the *Al* and cations content, influences their hydrophilicity, enhancing the attraction of water vapor. EFAl, surrounded by oxygen atoms in the framework, contributes negative charges, leading to strong interactions with water.^15,16^ Additionally, weaker interactions occur through pore/channel filling.^17^

This investigation aims to elucidate how the Si/Al ratio and compensating cations, individually and in combination, influence the hydrothermal stability of CHA zeolite in natural gas (NG) drying by thermal processes, extending and deepening previous studies.^18,19^ While earlier works established baseline aging conditions for CHA under TSA-relevant environments, the present study advances this knowledge by systematically isolating and evaluating additional aging stressors that are representative of industrial operation. Specifically, this investigation introduces a comparative assessment of hydrocarbon exposure modes, distinguishing between liquid-phase manual addition and controlled vapor-phase *n*-heptane exposure, and evaluates the effect of prolonged static aging under sustained high-temperature conditions. These aspects are particularly relevant to practical TSA units, where adsorbents may experience extended exposure to hot, hydrocarbon-containing streams without continuous regeneration. The experimental procedure was designed to simulate such harsh operating conditions, with key elements including: (1) manual addition and vapor-phase exposure to *n*-heptane; (2) better control of sample homogeneity in terms of elemental composition; and (3) maintenance of elevated temperatures during the aging procedure, including heating and cooling cycles. These methodological extensions, combined with detailed textural, surface, and adsorption analyses, enable a more comprehensive understanding of the mechanisms governing CHA aging beyond those previously reported. The results are organized into two sections: the first revisits the aging conditions originally proposed in the previous studies,^18,19^ serving as a reference framework, while the second provides an in-depth analysis of the effects of extended high-temperature and *n*-heptane vapor exposure over different aging durations using a CHA zeolite with a Si/Al ratio of approximately 2.0 in the sodium (Na) form.

## 2 Experimental Section

### 2.1 Adsorbent Syntheses

The synthesis of CHA in the Na-form with a Si/Al ratio of approximately 2.0 followed this procedure.^20^ Aluminum hydroxide (95%) supplied by Sigma-Aldrich was mixed with sodium hydroxide (97%) supplied by VWR Chemicals, potassium hydroxide (85%) supplied by VWR Chemicals, and tetramethylammonium hydroxide pentahydrate (97%) supplied by Sigma-Aldrich. The silica source, Ludox-LS-30 (30%, supplied by Aldrich), was then added. The final synthesis gel had the following composition: 0.0006 (TMA)_2_O: 6.67 Na_2_O: 2.2 K_2_O: 17.5 SiO_2_: Al_2_O_3_: 276 H_2_O. It is important to note that the atomic Si/Al ratio of the final adsorbent differs from that of the synthesis gel. During hydrothermal crystallization, aluminum is incorporated into the CHA framework with significantly higher efficiency than silicon, while a substantial fraction of the silica remains in solution, forms amorphous species, or is removed during postsynthesis washing. This gel was placed in a propylene bottle inside a preheated oven at 358 K for 4 h to facilitate crystallization. After crystallization, the solids were separated by vacuum filtration, washed with deionized water, and the filtrate was monitored until it reached neutrality. Following the synthesis, the sample underwent calcination at 823 K for 3 h to remove the remaining structure-directing agent (SDA). The samples were heated to 623 K at a ramp rate of 2.2 °C min^–1^ and held at this temperature for 2 h. They were then further heated to 823 K at a rate of 1.3 °C min^–1^ and maintained at this final temperature for 3 h. The resulting sample is labeled CHAc-SiAl2-Na, where “CHA” denotes the chabazite structure, “c” indicates crystal form, “SiAl2” represents the Si/Al ratio of approximately 2.0, and “Na” signifies sodium as the main compensating cation. In the next subsection, we will discuss the effects of temperature, CO_2_ and CH_4_ pressures, and humidity exposure on the samples. The sample labels include a suffix indicating their specific status: pristine (V) or aged material by different procedures (A35c, A20d, A30d, A45d, A60d).

### 2.2 Accelerated Aging Procedure

To simulate the aging of zeolite materials, a given amount of sample was subjected to conditions similar to those encountered in natural gas drying in offshore plants. These conditions include high temperatures (during desorption), high pressures (during adsorption), and the presence of humidity, heavy hydrocarbons, CH_4_, and CO_2_. The aging procedure used by Santiago et al. (2019)^18^ and Nascimento et al. (2021)^21^ involves the following steps: First, 25 g of the zeolite sample is placed in the bottom of a high-pressure Parr vessel (Model 3848 from Parr Company). Liquid *n*-heptane is added dropwise (0.6 mL per gram of sample), and the vessel is sealed. *n*-Heptane was selected as a model compound to represent heavy hydrocarbons in natural gas based on its representativeness within the C_7_^+^ fraction and on experimental considerations, allowing the investigation of key aging and fouling phenomena while avoiding the additional complexity and reduced reproducibility associated with multicomponent hydrocarbon mixtures. A moist N_2_ stream corresponding to water-saturated nitrogen (relative humidity ≈ 100%, *P*_H_2_O_ ≈ 42.5 mbar at 303 K) was used. The adsorbent was exposed to this stream for 24 h to ensure moisture equilibration prior to the experiments. The vessel is pressurized to 30 bar with a CO_2_/CH_4_ gas mixture (1:4 volume ratio), heated to 573 at 5 K min^–1^, maintained at 573 K for 3 h, and then cooled to 303 at 0.75 K min^–1^. This heating and cooling cycle is repeated 35 times, and the resulting sample is designated as (A35c). Alternatively, the procedure by Moura et al., 2022 ^19^ involves: placing 1 g of the sample in a hanging holder inside the measuring cell (chamber), degassing the chamber and connected tubing, and then exposing the sample to 1.0 mL of *n*-heptane and 0.5 mL of water per gram of sample for 24 h. The chamber is pressurized to 30 bar with a CO_2_/CH_4_ mixture (1:4 v/v) for 1 h. The temperature then increased to 573 at 5 K min^–1^, maintaining this temperature for 20, 30, 45, or 60 days. It is important to emphasize that the cyclic and static aging procedures were conceived to represent distinct operational parameters rather than to impose identical aging severities. The cyclic aging procedure, employing repeated heating–cooling cycles, is intended to mimic routine TSA operation. From a different perspective, the static procedure combines elevated temperatures with prolonged exposure at 573 K, intentionally accelerating aging processes in order to probe the upper-bound hydrothermal and chemical stability of the CHA zeolite. This temperature was selected based on conditions relevant to industrial regeneration operations. The resulting samples are designated as (A20d, A30d, A45d, A60d, respectively). A summary of all sample codes used in these studies is provided in Table 1.

**Table 1:** Samples and Descriptions

sample code	description	
CHAc-SiAl2-Na	-V	chabazite (CHA) with Si/Al = 2, Na^+^ as compensating cation. Pristine (not aged) material.
	-A35c	pristine material aged by the cyclic method: *n*-heptane addition, humidification with moist N_2_ (24 h), pressurized to 30 bar, heated to 573 K and cooled to 303 K (35 heating/cooling cycles).
	-Axd	pristine material aged by the static method: exposed to 1.0 mL *n*-heptane and 0.5 mL H_2_O per g sample (24 h), pressurized to 30 bar (1 h), then kept at 573 K for x = 20, 30, 45, or 60 days; (-A20d; -A30d; -A45d; -A60d).

### 2.3 Adsorbent Characterization

Pristine and aged adsorbent materials were characterized by X-ray diffraction (XRD) using a Philips X’Pert Pro MPD powder diffractometer (PANalytical). Diffractograms were collected in the 2θ range from 5° to 40° using Cu Kα radiation. X-ray photoelectron spectroscopy (XPS) analyses were performed on a Physical Electronics PHI 5700 spectrometer equipped with a nonmonochromatic Mg Kα X-ray source (1253.6 eV, 300 W, 15 kV) and a multichannel detector. Data acquisition and analysis were carried out using the PHI ACCESS ESCA-V6.0F software package, and binding energies were charge-referenced to adventitious carbon (C 1s at 284.8 eV).

The bulk elemental composition (C, H, N, S, and O) was determined using a LECO TruSpec Micro CHNSO elemental analyzer. Oxygen content was measured independently using the instrument’s dedicated high-temperature pyrolysis furnace operating at 1300 °C. The Si/Al ratio was obtained by solid-state ^29^Si and ^27^Al magic-angle spinning (MAS) NMR experiments performed on an AVANCE III HD 600 spectrometer (Bruker AXS). The Si/Al ratio estimated from the NMR data accounts for contributions from multiple silicon environments within the zeolite framework.

Nitrogen and carbon dioxide adsorption/desorption isotherms at 77 and 273 K, respectively (Autosorb iQ3, Quantachrome Instruments). To estimate the apparent area, the Brunauer-Emmett-Teller (BET) method was applied in the range that corresponds to the adsorption of a monolayer of the probe gas.^22^ The total pore volume was measured at a relative pressure of 0.99, considering all pores to be filled with liquid adsorbate. The specific surface area was calculated by applying the BET equation within an interval of relative pressure of 0.0001–0.03. The validity of the selected BET range was assessed using the Rouquerol consistency criteria.^23^ One of the most widespread equations to estimate the micropore volume was proposed by Dubinin–Radushkevich and was used in this work.^24^

Water vapor adsorption/desorption isotherms were measured in a high-accuracy gravimetric instrument IGA-002, by Hiden at 313 K and a pressure range from 10^–1^ to 70 mbar. Prior to the experiment, the samples were degassed under vacuum (10^–5^ mbar) and at 573 K for 10 h. Water vapor was then added to the sample, and the experimental data was adjusted using the Aranovich-Donohue Model–ADM (eq 1).^25^ This model takes into account the usual behavior at low pressures for microporous materials, represented by the Sips model^26^ and is combined with multilayer adsorption by water clustering.^27,28^

1 $$q_{\mathrm{eq}}=\frac{q_{\max}\cdot {\left(b\cdot P\right)}^{n}}{1+{\left(b\cdot P\right)}^{n}}\cdot \frac{1}{1-{\left(\frac{P_{o}}{P}\right)}^{e}}$$

## 3 Results and Discussion

### 3.1 CHA Sample Aged Under Different Conditions

Similar chabazite samples (CHAc-SiAl2-Na) were aged under two different conditions: (1) 35 heating–cooling cycles with manual addition of liquid *n*-heptane and (2) exposure to *n*-heptane vapor at high temperatures for 30 days. The diffractograms of the three aged samples show similar patterns, albeit with variations in the relative intensities of the reflection peaks (Figure 1). The main reflection peaks are observed in the 2θ range of 20 to 32° (20.4, 22.9, 24.7, 25.8, and 30.4°).^29^ All identified peaks match those of the pristine sample. The crystallinity of the aged samples, calculated as the sum of the areas under the main peaks between 20 and 32°, relative to the pristine sample, is presented in Table 2. The sample aged at consistently high temperatures experienced a significant reduction in crystallinity, approximately to 66% of the initial crystallinity. In contrast, the sample subjected to temperature cycles retained its crystallinity (Table 2). The comparison between the cyclic (A35c) and static (A30d) aging procedures also involves a substantial difference in cumulative exposure time at high temperature. The static method maintains the material at this temperature continuously for 30 days (∼720 h). In contrast, in the cyclic aging procedure, the residence time at high temperature is approximately 3 h per cycle, resulting in a total cumulative exposure of about 105 h over 35 cycles.

**Figure 1:**
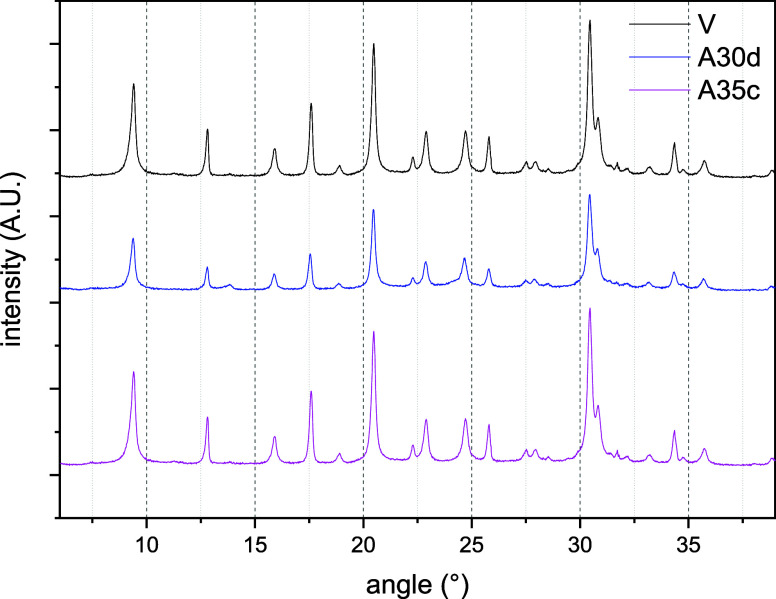
XRD patterns for CHAc-SiAl2-Na aged under different procedures (V, A35c, A30d).

**Table 2:** Materials Crystallinity Determined by XRD Diffractograms to the CHAc-SiAl2-Na (V, A35c, A30d)

sample	crystallinity [%]
CHAc-SiAl2–Na-V	100
CHAc-SiAl2-Na-A35c	98
CHAc-SiAl2-Na-A30d	66

The surface atomic concentrations, determined by XPS, for the pristine and aged samples are shown in Table 3. In general, the surface carbon content does not follow a clear trend in all solids, whereas the Si/Al ratios increase for any aged sample (Table 4). It may be that carbon deposition preferentially occurs at the stronger acid sites (Al–O), thus covering Al atoms on the surface and leading to an apparent increase of the Si/Al ratio. The increase in the surface Si/Al ratio should therefore not be attributed exclusively to coke masking of aluminum sites. Hydrothermal aging is known to promote partial surface dealumination and/or the formation of silica-enriched surface layers in CHA-type zeolites. The observed Si/Al increase is thus interpreted as the combined effect of aluminum loss or migration at the surface and attenuation of Al photoelectron signals due to subsurface carbonaceous deposits.^30^

**Table 3:** Surface Chemical Composition Determined by XPS Analysis (% Surface Atomic Concentration) for the CHA_C_-SiAl2-Na- (V, A35c, A30d)

sample	C 1*s*	O 1*s*	Al 2p	Si 2p	Na 1s	K 2p
CHAc-SiAl2–Na-V	17.76	59.05	5.53	11.88	5.18	0.59
CHAc-SiAl2-Na-A35c	23.27	55.30	3.94	10.01	7.01	0.46
CHAc-SiAl2-Na-A30d	15.59	61.52	4.17	11.87	6.25	0.59

**Table 4:** Atomic Si/Al Ratios, as Determined by XPS Analysis for the CHA_C_-SiAl2-Na- (V, A35c, A30d)

sample	Si/Al atomic ratio
CHAc-SiAl2–Na-V	2.2
CHAc-SiAl2-Na-A35c	2.6
CHAc-SiAl2-Na-A30d	3.0

CHN elemental analysis (Table 5) shows an increase in carbon content in the bulk of the aged samples compared to the fresh sample. When comparing the two aging methods, the sample aged with *n-heptane* vapor exposure exhibited a smaller increase in carbon content, which may be related to partial deterioration of textural properties and the reduction of water adsorption,^31^ as discussed in the following sections.

**Table 5:** Elemental CHN Analysis for the CHA_C_-SiAl2-Na- (V, A35c, A30d)

sample	C [%]	H [%]	N [%]
CHAc-SiAl2–Na-V	<0.3	1.8 ± 0.3	<0.3
CHAc-SiAl2-Na-A35c	3.0 ± 1.8	3.1 ± 1.5	2.5 ± 2.1
CHAc-SiAl2-Na-A30d	0.9 ± 0.3	1.9 ± 0.3	<0.3

When liquid *n*-heptane was dropwise added to the samples, the carbon content varied significantly at different points in the same batch. Considerable variability was found when analyzing different samples across several measurements. This variability is most likely associated with the uneven application and mixing of the hydrocarbon on the material surface. To ensure representativeness of the entire adsorbent load, triplicate analyses were performed using aliquots collected from different spatial positions within each batch, and the corresponding standard deviations for each element were calculated and are reported in Table 5.

The detected nitrogen signals are not attributed to framework incorporation or chemically bonded nitrogen species in the CHA structure, as chabazite does not contain nitrogen in its framework and no nitrogen-containing reagents were employed during synthesis or aging. Therefore, the low and occasionally variable nitrogen contents observed in the CHN analyses are most plausibly related to residual contaminants, weakly trapped species, or instrumental and analytical uncertainty at concentrations close to the detection limit.

Although XPS and CHN analyses seem to indicate opposite trends in carbon content, these observations are not contradictory, as they reflect the different spatial sensitivities of the two techniques. XPS probes only the outermost surface layers, whereas CHN provides an average bulk composition of the material. The relatively higher surface carbon detected by XPS in the pristine sample is mainly attributed to adventitious carbon and residual organic species originating from synthesis, handling, or ambient exposure. Such species are readily detected by surface-sensitive techniques and are partially removed during hydrothermal aging at elevated temperature. In contrast, the increase in bulk carbon content observed by CHN in aged samples indicates the accumulation of carbonaceous species within internal and near-surface domains of the zeolite, including subsurface regions, pore mouths, and intercrystalline voids that are not fully accessible to XPS.

NMR analyses (Table 6) indicate that the Si/Al ratio remains consistent in the aged materials. However, XPS analyses show an increased Si/Al ratio on the external surface of the material (Table 4), highlighting a clear surface–bulk discrepancy. This behavior can be rationalized by the contribution of complementary mechanisms acting preferentially at the zeolite surface during aging. One plausible explanation is the preferential deposition of carbonaceous species on aluminum-associated acidic sites. Literature reports indicate that Brønsted acid sites enhance zeolite deactivation and promote adsorption and activation of central C–C bonds, favoring the formation and accumulation of carbon deposits at the surface.^32,33^ Such deposits may partially mask surface aluminum species, leading to an artificially higher Si/Al ratio measured by surface-sensitive XPS, while the bulk composition probed by NMR remains unaffected. Additionally, hydrothermal treatment at elevated temperatures may induce aluminum migration or redistribution, either within the framework or toward extra-framework positions.^19,34–36^ This process can result in local depletion of framework aluminum near the external surface without significantly altering the overall bulk Si/Al ratio.

**Table 6:** Si/Al Ratio by NMR Analyses for the CHA_C_-SiAl2-Na- (V, A35c, A30d)

sample	Si/Al ratio
CHAc-SiAl2–Na-V	2.1
CHAc-SiAl2-Na-A35c	2.1
CHAc-SiAl2-Na-A30d	2.0

The nitrogen isotherms at 77 K for the pristine CHA sample show a characteristic behavior of microporous solids with good adsorbent–adsorbate interaction, or type I isotherms, up to a relative pressure of 0.8, according to the IUPAC classification^37^ (Figure 2). In this pressure range, the measured adsorbed concentrations agree with the literature data for CHA zeolites.^38^ Beyond a relative pressure of 0.8, nitrogen uptake rises steeply, which may be due to condensation in the interstitial voids between particles. In addition, a decrease in uptake of N_2_ is observed to varying degrees in aged materials. Santiago et al. (2019)^18^ and Moura et al. (2022)^19^ have previously noted that the degradation of textural properties is closely related to the partial pressure of the hydrocarbon. The nonmanual addition of *n*-heptane, which allows homogeneous vapor-phase preadsorption of water and *n*-heptane, appears to be an improvement over manual liquid addition. Comparison of the BET specific surface area, DR micropore volume and total pore volume from N_2_ adsorption isotherms at 77 K (Table 7) indicates that the aging effects are comparable for samples A35c and A30d, suggesting severe pore blockage after the aging process.

**Figure 2:**
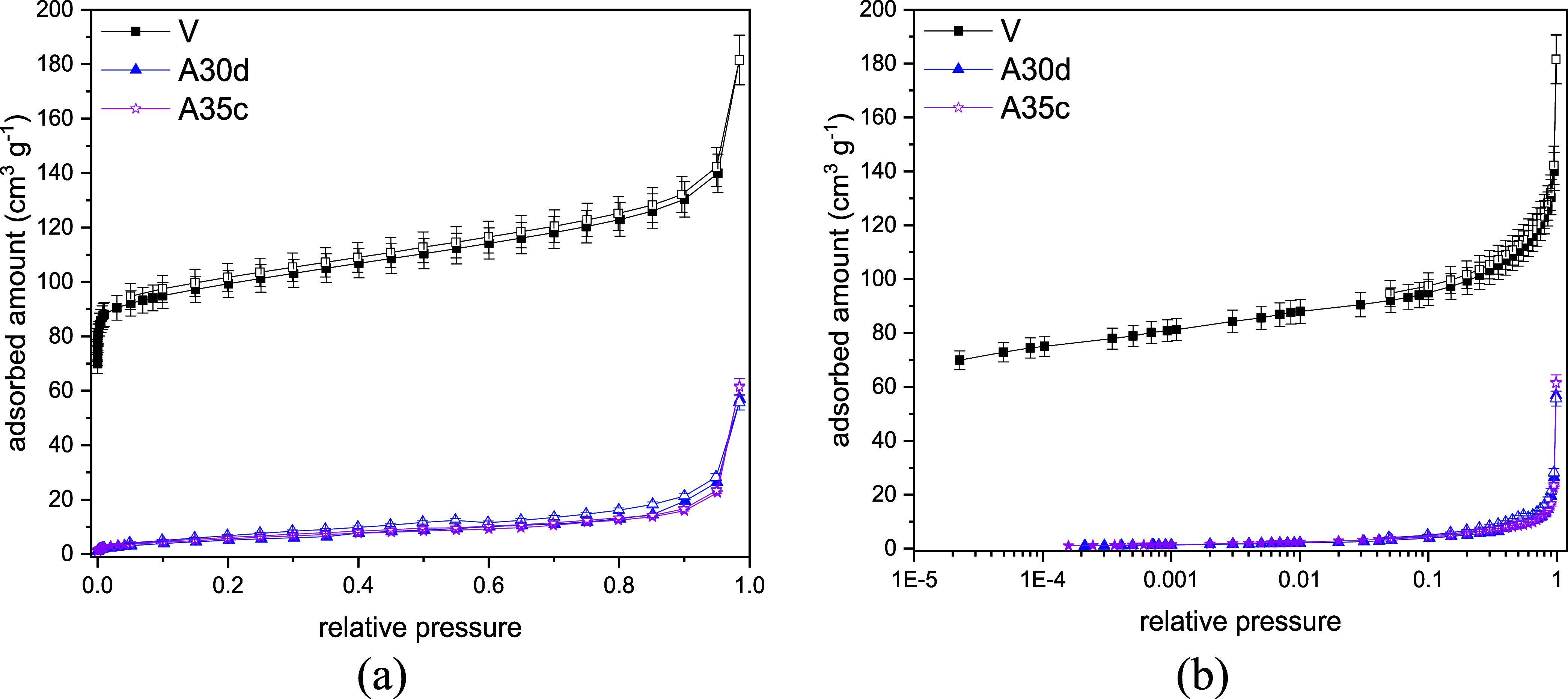
Nitrogen adsorption isotherms at 77 K in linear (a) and logarithmic (b) scales for CHA_C_-SiAl2-Na- (V, A35c, A30d).

**Table 7:** Apparent BET Area, Pore Volume, Adsorption Capacity and Micropore Volume for the CHA_C_-SiAl2-Na- (V, A35c, A30d)

	from N_2_ isotherms at 77 K			from CO_2_ isotherms at 273 K	
sample	apparent BET area [m^2^ g^–1^]	micropore volume [cm^3^ g^–1^]	total pore volume [cm^3^ g^–1^]	max adsorption capacity^a^ [cm^3^ g^–1^]	micropore volume [cm^3^ g^–1^]
CHAc-SiAl2–Na-V	352	0.132	0.24	110.7	0.225
CHAc-SiAl2-Na-A35c	20	0.007	0.09	63.6	0.138
CHAc-SiAl2-Na-A30d	24	0.008	0.12	74.9	0.156

a At a relative pressure of 0.029 References 22,24

For CO_2_ adsorption at 273 K, the isotherms exhibit a type I shape (Figure 3), according to the IUPAC classification.^37^ Porosity analysis from N_2_ and CO_2_ adsorption isotherms at 77 and 273 K, respectively (Table 7), shows that the different kinetic diameters of the probe molecules and the higher temperature in the CO_2_ experiments enable the molecules to access the microporosity more effectively, revealing a significant micropore volume. After severe pore blocking, micropore volumes derived from N_2_ adsorption provide a more sensitive indication of structural obstruction. In contrast, CO_2_ adsorption at 273 K and pressures up to 1 bar cannot fully access pores larger than approximately 15 Å, which may lead to an underestimation of pore blockage effects.^37^ Accordingly, CO_2_-derived micropore volumes for samples CHAc-SiAl2-Na-A30d and CHAc-SiAl2-Na-A35c do not indicate pronounced pore blocking, whereas N_2_-based micropore analysis reveals a more significant reduction in accessible microporosity. The pristine sample has a micropore volume of 0.225 cm^3^ g^–1^, which is reduced in the aged samples, likely due to the effects of high temperatures and hydrocarbon exposure. Hydrocarbon exposure under high-temperature conditions promotes the formation and accumulation of carbonaceous species on external surfaces and within internal domains of the material, primarily as a result of hydrocarbon transformation and pore-mouth obstruction rather than direct diffusion of intact molecules into CHA micropores. Additionally, it provides a clearer view in the low-pressure range, offering a more detailed analysis of how the aging procedures impact the stronger adsorption sites.

**Figure 3:**
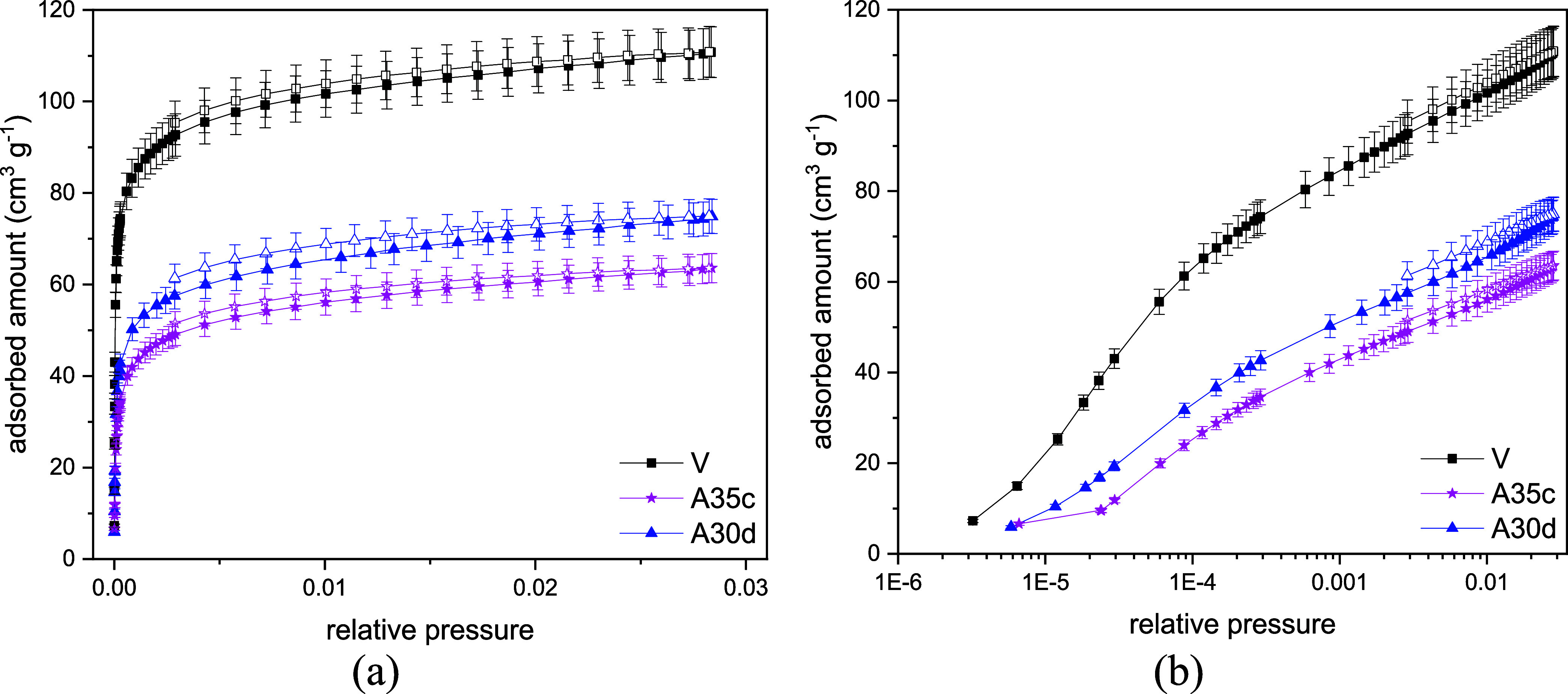
Carbon dioxide adsorption isotherms at 273 K in linear (a) and logarithmic (b) scales for CHA_C_-SiAl2-Na- (V, A35c, A30d).

The water vapor isotherms at 313 K (Figure 4) also show that the adsorption capacities of the aged materials are lower than those measured for the pristine sample, although the differences in adsorption are significantly more modest than those observed for N_2_ and CO_2_ adsorption (Figures 2 and 3). All isotherms show a gradual increase in the low-pressure range, up to 5 mbar, indicating strong adsorbate–adsorbent interactions that persist despite the aging method. In the pressure range 5 to 40 mbar, the isotherms reach a plateau, which is lower for the aged samples and similar for those aged at consistently high temperatures. Beyond 40 mbar, there is a marked rise in the high-pressure region. Sample A35c exhibits the lowest microporosity among the aged samples (Table 7) and also the highest carbon content (Table 5). This carbon deposition appears to clog some of the zeolite pores, as indicated by the very low N_2_ uptake in the adsorption isotherms (Figure 2). However, some strong adsorption sites appear to be retained within the micropores, as demonstrated by the CO_2_ isotherms (Figure 3) and further confirmed by the water isotherms in the low-pressure range (Figure 4(b)). In the sample aged by exposure to *n*-heptane vapor, carbon appears to be deposited in the stronger adsorption sites, reducing CO_2_ and H_2_O uptake in the low-pressure range, despite a modest *C* content as compared to the sample aged by manual *n-*heptane liquid addition. To achieve a comparable level of degradation in aged samples: (1) hydrocarbon vapor exposure should be extended for a longer duration, and (2) for samples aged under temperature cycling, the cycling duration should be increased. The Aranovich-Donohue model (ADM) provided a satisfactory fit for all isotherms across the entire pressure range (Figure 4). The ADM parameters (Table 8) align with previous observations.

**Figure 4:**
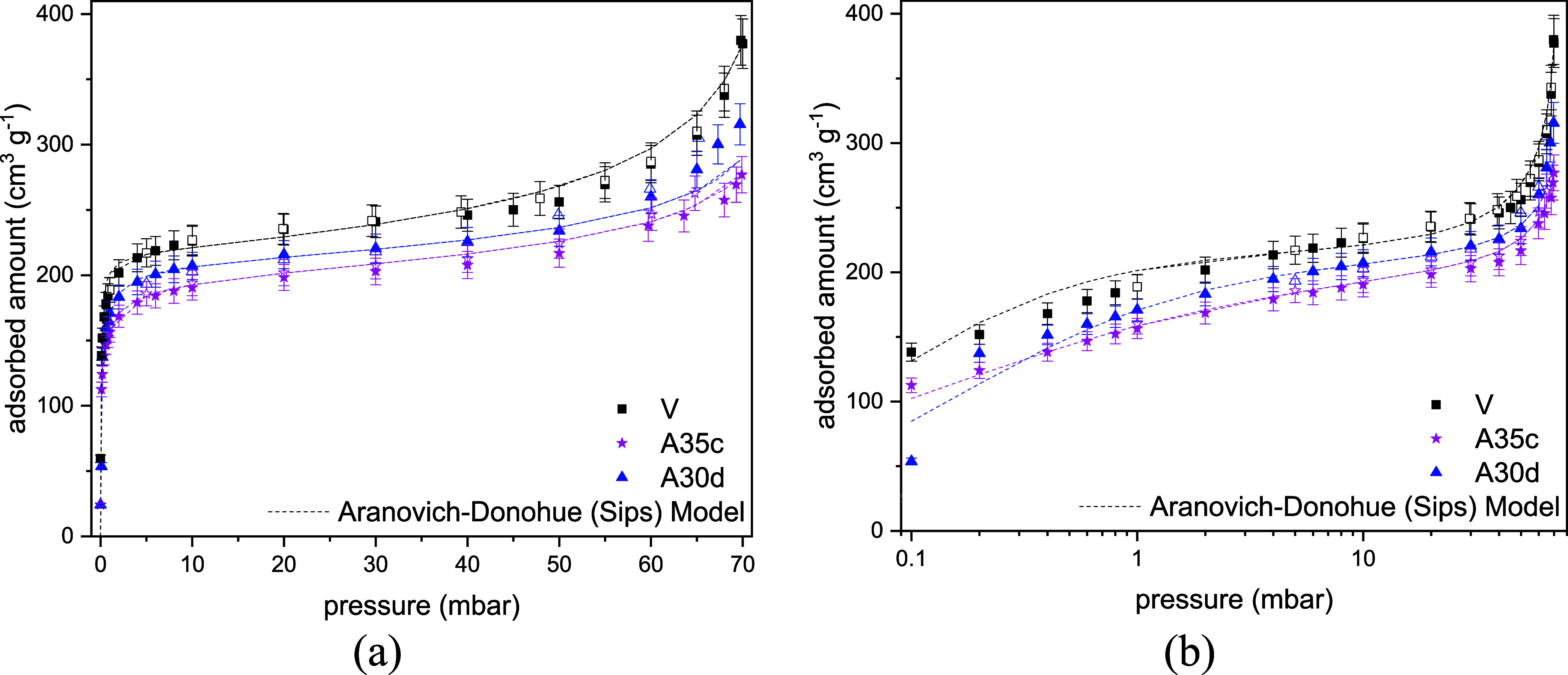
Water adsorption isotherms at 313 K in linear (a) and logarithmic (b) scales for CHA_C_-SiAl2-Na- (V, A35c, A30d). The dashed curves stand for the Aranovich-Donohue model fits.

**Table 8:** Parameters in Aranovich-Donohue Model Fitting for H_2_O Isotherms at 313 K for CHA_C_-SiAl2-Na- (V, A35c, A30d)

	parameters			
sample	*q*_max_ [mg g^–1^]	*b* [mbar^–1^]	*n*	*e*
CHAc-SiAl2–Na-V	217.41	16.07	0.90	0.19
CHAc-SiAl2-Na-A35c	207.29	9.54	0.52	0.11
CHAc-SiAl2-Na-A30d	210.69	6.10	0.80	0.11

### 3.2 In-Depth Assessment of Aging Effects by High-Temperature

In this second section, we will undertake a detailed analysis of the material subjected to aging under conditions including exposure to water and *n*-heptane vapor, in conjunction with sustained high temperatures. The X-ray diffraction patterns of the pristine and aged CHA samples (Figure 5) exhibit similar peak positions, though with varying intensities. The overall peak order remains consistent, suggesting that the structural features are comparable despite the differences in peak magnitude.^3,29^ The primary reflection peaks in the 2θ range of 20 to 32° (Miller Index), specifically at 20.4° (2 0 −1), 22.9° (2 1 −1), 24.7° (2 1 1), 25.8° (2 0 −2), and 30.4° (2 2 −1), align with the CHA pattern and those of the pristine sample. These peaks are characteristic of the CHA crystal structure.^39^ Materials aged with *n*-heptane vapor exposure and high temperatures for 20, 30, 45, and 60 days (designated as A20d, A30d, A45d, and A60d, respectively) display similar diffraction patterns (Figure 5). However, the intensity of the peaks decreases with prolonged aging, which reflects a reduction in the material’s crystallinity. Crystallinity values, calculated as the ratio of the sum of areas under the main peaks in the 2θ range from 20 to 32° to the sum of areas under the same peaks for the pristine sample, are provided in Table 9. Aged samples show a decrease in crystallinity with longer aging periods, with the most significant deterioration occurring within the first 30 days. After 45 days, the rate of degradation levels off.

**Figure 5:**
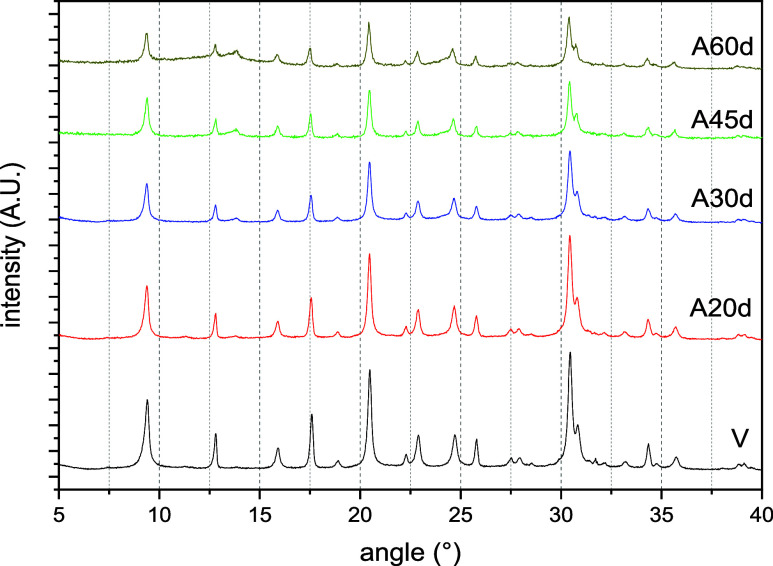
XRD diffractograms for the CHAc-SiAl2-Na series aged by *n*-heptane vapor exposure at high temperature.

**Table 9:** Materials Crystallinity from XRD Diffractograms to the CHAc-SiAl2-Na Series Aged by *n*-heptane Vapor Exposure at High Temperature

sample	crystallinity [%]
CHAc-SiAl2–Na-V	100
CHAc-SiAl2-Na-A20d	97
CHAc-SiAl2-Na-A30d	66
CHAc-SiAl2-Na-A45d	58
CHAc-SiAl2-Na-A60d	57

The main elements present in the surface of both pristine and aged materials, as identified by X-ray photoelectron spectroscopy (XPS), are summarized in Table 10 along with their respective Binding Energies (BE). The BE for the high-resolution C 1*s* core level spectra corresponds to C–C and C–O bonds, while the BE for Si 2p and Al 2p spectra reflect the tetrahedral coordination typical of aluminosilicates. The BE for O 1*s* also corresponds to the oxygen atoms in aluminosilicates.^40^ The mass compositions of Si, Al, Na, and K on the surface are presented as stacked bar charts in Figure 6. Although the *C* and *O* contents vary erratically and show no clear correlation with the aging process, the Si/Al ratio increases by nearly 30% on the surface with prolonged aging (Table 11). This increase in Si concentration is possibly due to the higher thermal stability of Si–O–Si bonds, which contributes to the increased Si content on the surface^13,14^ (Figure 6). It is possible that carbon deposits, either from strongly adsorbed *n-heptane* or coke, preferentially surrounding the Al-centered tetrahedra, which are acidic sites. This would cover these sites and enhance the Si/Al ratio.^19,30^ Additionally, XPS results (Table 10) associated with CHN analysis (Table 5) suggest that carbon deposition occurs both inside and on the surface of the samples. Aging appears to promote the migration of Al to the interior of the material.

**Figure 6:**
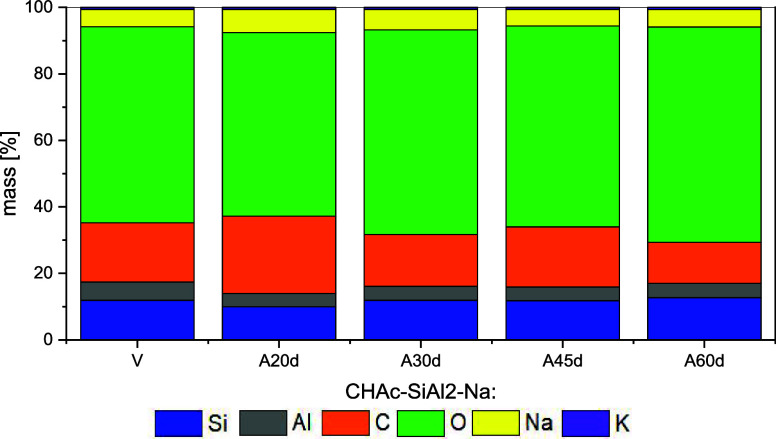
Surface chemical composition (% mass concentration) determined by XPS analyses for the CHA_C_-SiAl2-Na- (V, A20d, A30d, A45d, A60d).

**Table 10:** Binding Energy Values (eV) as Detected by XPS for the CHA_C_-SiAl2-Na Series Aged by *n*-heptane Vapor Exposure at High Temperature

sample	Si 2p	Al 2p	C 1*s*	O 1*s*	Na 1*s*	K 2p_3/2_
CHAc-SiAl2–Na-V	102.6	74.6	285.0 286.6	531.8	1072.7	294.6
CHAc-SiAl2-Na-A20d	102.6	74.3	284.8 286.6	531.8	1072.4	294.5
CHAc-SiAl2-Na-A30d	102.8	74.5	284.6 286.6	531.9	1072.6	294.6
CHAc-SiAl2-Na-A45d	102.7	74.4	284.8 286.1	531.9	1072.6	294.3
CHAc-SiAl2-Na-A60d	102.8	74.4	284.8 285.9	532.0	1072.6	294.4

**Table 11:** Obtained Si/Al Atomic Ratio Determined by XPS for the CHA_C_-SiAl2-Na Series Aged by *n*-Heptane Vapor Exposure at High Temperature

sample	Si/Al atomic ratio
CHAc-SiAl2–Na-V	2.2
CHAc-SiAl2-Na-A20d	2.6
CHAc-SiAl2-Na-A30d	3.0
CHAc-SiAl2-Na-A45d	3.0
CHAc-SiAl2-Na-A60d	3.1

CHN elemental analyses of the pristine sample and those aged for 30 and 60 days, reveal a notable increase in bulk carbon content (Table 12). This increase suggests a significant carbon accumulation, which, as reported by Karge (1991),^41^ is associated with coke formation in zeolites. The carbon content observed in the samples aged for a shorter period (CHAc-SiAl2-Na-A30d) supports the hypothesis that carbon buildup, likely due to coke formation induced by high temperatures, occurs within the solid.

**Table 12:** Elemental CHN Analysis for the Pristine (V) and Aged by *n*-Heptane Vapor Exposure at High Temperature for 30 Days (A30d) and 60 Days (A60d)

sample	C [%]	H [%]	N [%]
CHAc-SiAl2–Na-V	<0.3	1.8 ± 0.3	<0.3
CHAc-SiAl2-Na-A30d	0.9 ± 0.3	1.9 ± 0.3	<0.3
CHAc-SiAl2-Na-A60d	3.1 ± 0.3	1.9 ± 0.3	0.8 ± 0.3

The N_2_ adsorption isotherms for any of the aged samples (Figure 7), regardless of the duration of the procedure, show a drastic decrease in uptake as compared to the pristine sample, comparable to the isotherms of nonporous solids. The aging effect is dramatic, particularly for relative pressure below 0.1. When aged solids are compared to each other above a relative pressure of 0.1, the adsorption capacity decreases as the number of aging days increases (Figure 7). This acute reduction in N_2_ adsorption is probably due to carbon deposition detected by CHN elemental analysis (Table 12). Carbon deposition may occupy/block pores, which is corroborated by the equally pronounced drop in total pore volume, as shown in Table 13. For the aged samples, the specific apparent BET area, DR micropore volume and total pore volume values obtained from the N_2_ isotherms at 77 K are similar, irrespective of the number of days of the aging procedure (Table 13). For this reason, isotherms using another probe gas or temperature may provide further information on the impact of increasing aging severity on textural properties.

**Figure 7:**
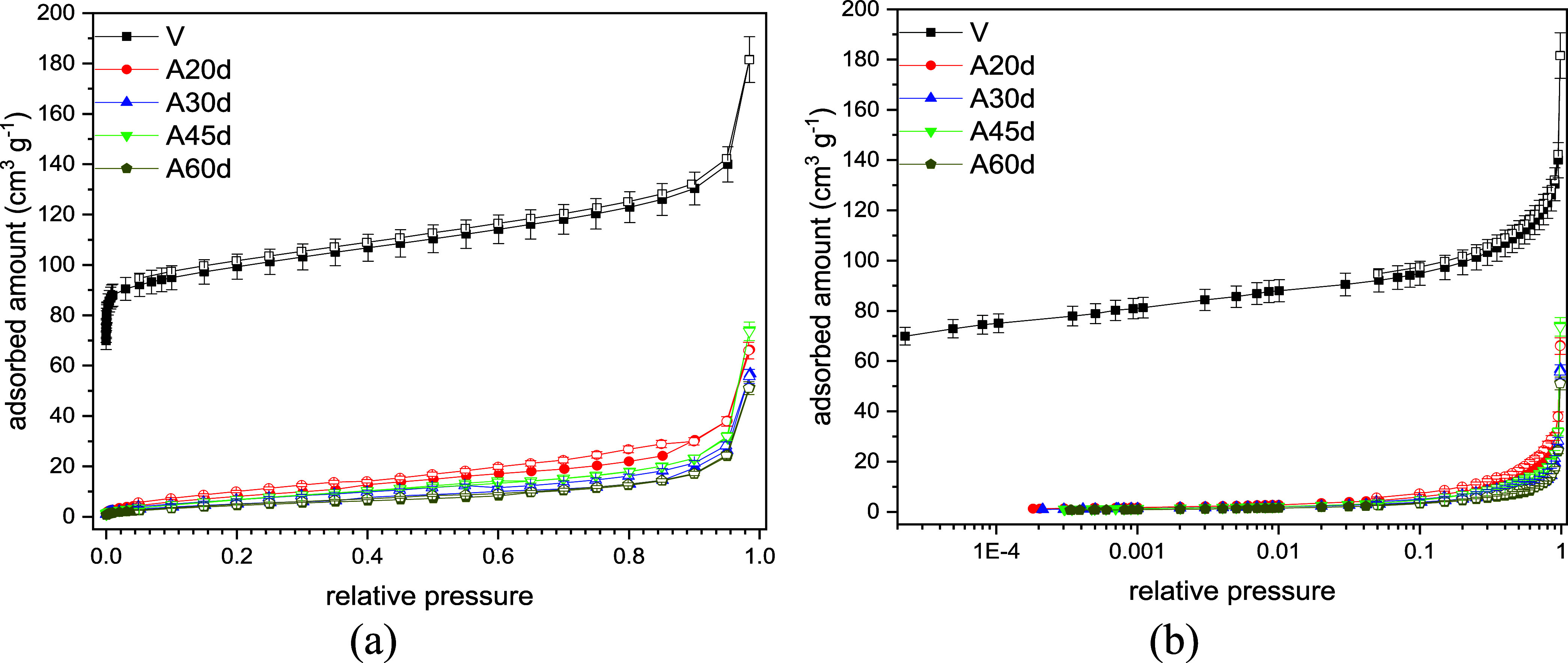
Nitrogen adsorption isotherms at 77 K for the CHA_C_-SiAl2-Na series aged by *n*-heptane vapor exposure at high temperature in linear (a) and logarithmic (b) scales.

**Table 13:** Apparent BET Area and Pore Volume from N_2_ Isotherms at 77 K for the CHA_C_-SiAl2-Na Series Aged by *n*-Heptane Vapor Exposure at High Temperature

sample	apparent BET area [m^2^ g^–1^]	micropore Volume DR [cm^3^ g^–1^]	total pore volume [cm^3^ g^–1^]
CHAc-SiAl2–Na-V	352	0.132	0.239
CHAc-SiAl2-Na-A20d	29	0.011	0.103
CHAc-SiAl2-Na-A30d	24	0.008	0.120
CHAc-SiAl2-Na-A45d	25	0.008	0.115
CHAc-SiAl2-Na-A60d	17	0.006	0.079

The CO_2_ isotherms at 273 K for the pristine and aged samples are illustrated in Figure 8. Due to its low thermal energy at 77 K, N_2_ is unable to overcome diffusion barriers imposed by narrowed pore entrances or partially blocked micropore openings, whereas CO_2_ at 273 K can still access a significant fraction of the internal microporosity. This kinetic gating effect suggests that the internal pore network remains largely intact after aging, albeit with restricted accessibility. Unlike N_2_ isotherms, there is now a clear correlation between CO_2_ uptake and aging severity (Figure 8). When checking the isotherms on a log scale (Figure 8 (b)), the progressive decrease in uptake suggests that aging leads to pore clogging, to an extent where only CO_2_ may diffuse through them. The pristine sample has a maximum adsorption capacity of 110.7 cm^3^ g^–1^, while the most severely aged sample (A60d) has less than 30% of said capacity. Similar to the trend observed in crystallinity (Table 9), the aging rate seems to slow down after 45 days, when prolonging the aging period no longer has a massive impact on the adsorption capacity. The micropore volume of the samples was estimated by the Dubinin–Radushkevich method using CO_2_ isotherms at 273 K^24^ and the values are condensed in Table 14. Comparing the total pore volume, estimated from N_2_ isotherms (Table 13), and the micropore volume estimated from CO_2_ isotherms (Table 14), the same trends are confirmed, although N_2_ at 77 K does not have access to the full range of pores, especially the ultramicropores produced by carbon deposition during aging.

**Figure 8:**
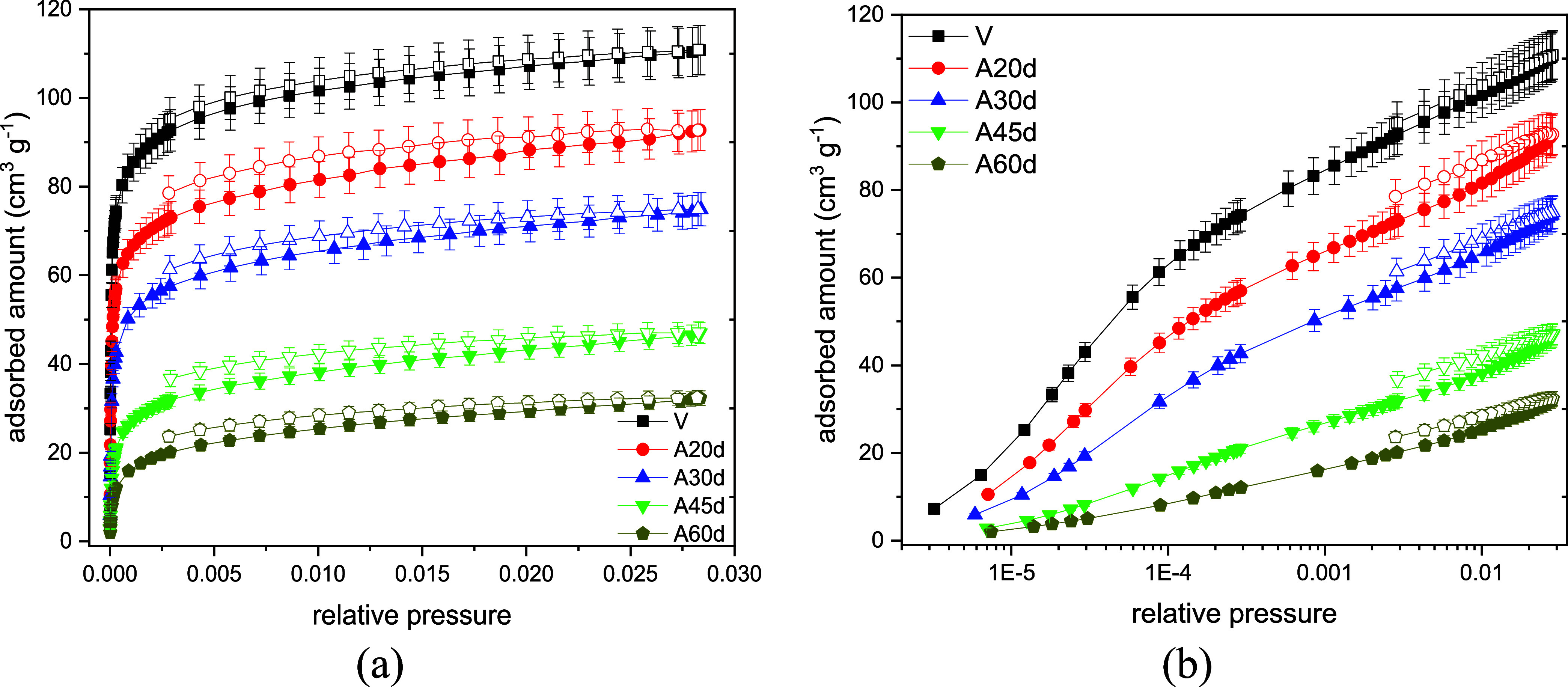
Carbon dioxide adsorption isotherms at 273 K for the CHA_C_-SiAl2-Na series aged by *n*-heptane vapor exposure at high temperature in linear (a) and logarithmic (b) scales.

**Table 14:** Adsorption Capacity and Micropore Volume from CO_2_ Isotherms at 273 K for the CHA_C_-SiAl2-Na Series Aged by *n*-Heptane Vapor Exposure at High Temperature

sample	maximum adsorption capacity^a^ [cm^3^ g^–1^]	micropore volume [cm^3^ g^–1^]
CHAc-SiAl2–Na-V	110.7 (100%)	0.225
CHAc-SiAl2-Na-A20d	92.7 (83.7%)	0.182
CHAc-SiAl2-Na-A30d	74.9 (67.7%)	0.156
CHAc-SiAl2-Na-A45d	47.0 (42.4%)	0.095
CHAc-SiAl2-Na-A60d	32.3 (29.2%)	0.072

a At a relative pressure of 0.029

In Figure 9, water adsorption isotherms are illustrated for the pristine sample and those aged for 30 and 45 days. The experimental data were fitted by the Aranovich-Donohue Model (ADM),^25^ which considers micropore filling according to the Sips model^26^ multiplied by a term representing clustering or condensation at larger voids/defects in the zeolite structure (eq 1). The shape of isotherms comprises a sharp increase in the low-pressure range, which confirms the high adsorbent–adsorbate affinity. The interactions of water molecules and the zeolite structure come from the zeolite affinity for polar molecules, induced by the unbalanced charges generated by AlO_4_^–^.^42,43^ As expected, the pristine material presents a higher adsorption capacity than the aged samples (A30d and A45d) in the entire pressure range. This decrease in water uptake (Figure 9) between the fresh and aged samples is associated with the reduction in accessible porosity in the aged samples, presented by the N_2_ isotherms at 77 K (Figure 7) and CO_2_ at 273 K (Figure 8) isotherms, probably caused by carbon deposition. Nevertheless, the deterioration in water uptake for the aged samples was comparatively less severe than that observed in the textural properties, indicating that the zeolite retains most of its hydrophilic character, despite the impact of hydrothermal aging on other properties. From an industrial TSA perspective, these results indicate that aging-induced structural degradation does not necessarily translate into a proportional loss of dehydration capacity, as the water adsorption performance is moderately affected under process-relevant conditions. When analyzing the high-pressure zone (pressures close to the saturation of water at 313 K), the difference in adsorption capacity between the samples becomes greater. The pristine sample presents a steeper rise in uptake above 50 mbar compared to the aged materials, that could be associated with a higher density of larger pores (voids or interstitial defects).

**Figure 9:**
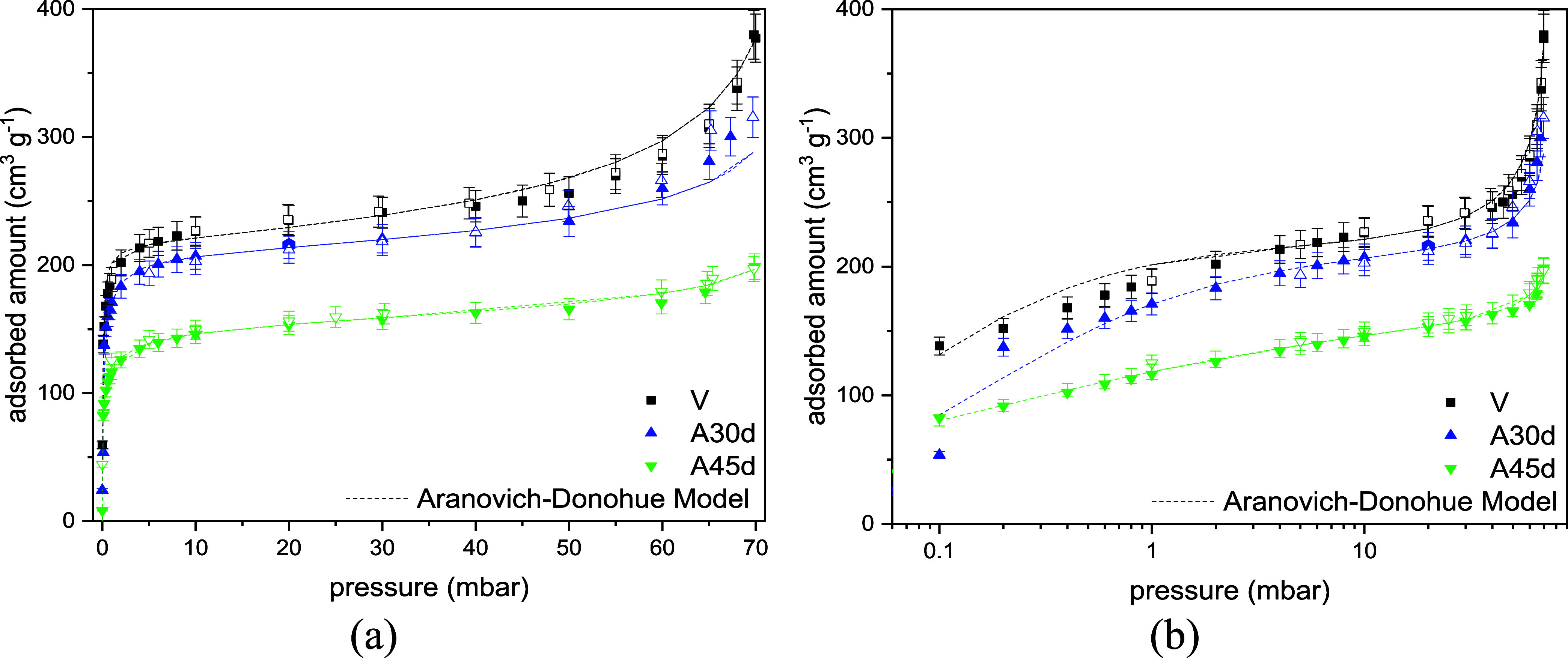
Water adsorption isotherms at 313 K for the CHAc-SiAl2-Na-(V, A30d, A45d) aged by *n*-heptane vapor exposure at high temperature in linear (a) and (b) logarithmic scales.

The isotherm fitting parameters of the Aranovich-Donohue model (ADM) are summarized in Table 15. The parameter representing the water-zeolite interaction (*b*) decreases by almost an order of magnitude compared to the pristine sample. This decrease in adsorbent/adsorbate affinity likely reflects the impact of carbon deposition (Table 12), which diminishes the availability of acid sites on the zeolite that strongly bind water. Additionally, the parameter describing surface homogeneity (*n*) decreases with aging, indicating changes in the energetic topology of the zeolite as a result of deactivation.

**Table 15:** Parameters in Aranovich-Donohue Model Fitting for Water Adsorption Isotherms at 313 K for CHAc-SiAl2-Na-(V, A30d, A45d) Aged by *n*-Heptane Vapor Exposure at High Temperature

	parameters			
sample	*q*_max_ [mg g^–1^]	*b* [mbar^–1^]	*n*	*e*
CHAc-SiAl2–Na-V	217.41	16.07	0.90	0.19
CHAc-SiAl2-Na-A30d	210.69	6.10	0.80	0.11
CHAc-SiAl2-Na-A45d	168.30	7.97	0.41	0.08

## 4 Conclusions

This study demonstrates that maintaining high temperatures during the aging process leads to a notable degradation of the zeolite’s crystalline structure. Prolonged aging correlates with greater degradation of various properties, suggesting that *n-heptane*, when applied in the vapor phase, adsorbs in the inner and outer pores of the zeolite. This adsorption is linked to the observed deterioration in textural characteristics, which are directly associated with a high carbon content in the bulk material. Aging durations of 20–30 days result in the most pronounced alterations in porosity and crystallinity, although these changes are less severe compared to those induced by the direct hydrocarbon addition. High-temperature conditions contribute to a more substantial loss of crystallinity. Additionally, samples subjected to heating–cooling cycles exhibit a less homogeneous composition, as evidenced by CHN analysis. These findings underscore the impact of aging conditions on zeolite stability and highlight the importance of controlling aging parameters to preserve material integrity.

## List of Abbreviations

ADM: Aranovich-Donohue Model
CHA: Chabazite materials
CHN: Carbon – Hydrogen – Nitrogen
BE: Binding Energies
BET: Brunauer – Emmett – Teller
EFAl: Extra-Framework Aluminum
IUPAC: International Union of Pure and Applied Chemistry
NMR: Nuclear Magnetic Resonance
SDA: Structure-Directing Agent
TMA: *N*,*N*,*N*-trimethyl-1-adamantylammonium
TSA: Temperature Swing Adsorption
XPS: X-ray Photoelectron Spectroscopy
XRD: X-ray Diffraction
